# Supplementary material for: Dynamical Control of Coulomb Interactions and Hubbard Bands in Monolayer 1T-TaS2
Source: Nano Lett. 2026 Apr 8;26(15):4973–8. doi: 10.1021/acs.nanolett.5c05443 (PMC13107445; doi:10.1021/acs.nanolett.5c05443)
Supplement: Supplementary file 1 [file nl5c05443_si_001.pdf]

Supplementary material for 'Dynamical control of Coulomb interactions and Hubbard bands in monolayer 1T-TaS_2'

Niklas Notter and Markus Aichhorn
Institute of Theoretical and Computational Physics,
TU Graz, NAWI Graz, Petersgasse 16, 8010 Graz, Austria

Anna Galler
Institute of Theoretical and Computational Physics, TU Graz,
NAWI Graz, Petersgasse 16, 8010 Graz, Austria and
Max Planck Institute for the Structure and Dynamics of Matter,
Center for Free Electron Laser Science, Luruper Chaussee 149, 22761 Hamburg, Germany

(Dated: April 1, 2026)

In this supplementary material, we present technical details concerning the calculations presented in the main text, as well as additional results underlining our main findings.

I. DFT CALCULATIONS AND LATTICE RELAXATIONS

The DFT calculations for the 1T-TaS_2 monolayer were performed using the Vienna ab initio simulation package (VASP)[1–3] (version 6.4.3), by employing the Perdew, Burke, and Ernzerhof (PBE)[4] exchange-correlation functional, and projector augmented wave (PAW) pseudopotentials[5,6]. We used a lattice constant of 12.20 Å and a Γ-centered $8 \times 8 \times 1$ k-point mesh for the $\sqrt{13} \times \sqrt{13}$ supercell. To avoid interaction between periodic images, a vacuum layer of 20 Å was included. All calculations were performed using a cutoff energy of 400 eV.

The lattice structure of the Star-of-David (SoD) charge density wave (CDW) phase was obtained by relaxing the $\sqrt{13} \times \sqrt{13}$ supercell. The relaxations were performed in VASP using the conjugate-gradient method, with the total energy and residual atomic forces converged to within 10^{-8} eV and 0.01 eV/Å, respectively, with atomic positions relaxed at fixed cell volume. Gaussian smearing with a width of 0.05 eV was used during relaxation, followed by a static calculation with the tetrahedron method and Blöchl corrections for accurate total energies. The energy–volume data were fitted with the Birch–Murnaghan equation of state, and a final relaxation was performed at the optimized volume.

The amplitude of the SoD CDW was quantified as $a = (d - d_{\mathrm{eq}})/d_{\mathrm{eq}}$, where d is the distance between the central (A) and outer Ta atoms (C) in the SoD, and d_{eq} is the corresponding distance in the fully distorted (equilibrium) CDW phase. Atomic positions for a frozen CDW amplitude a were generated by linear interpolation and extrapolation of the fully distorted SoD CDW structure and the undistorted p-$3m1$ structure.

II. DFT TOTAL ENERGY CALCULATIONS

In Fig. S1, we present the DFT total energy as a function of the CDW amplitude a. As expected, the minimum occurs at the equilibrium CDW distortion ($a = 0\%$). For small amplitudes ($|a| < 2\%$), the energy profile is well described by a harmonic potential, whereas deviations from harmonic behaviour become evident at larger amplitudes, indicating the onset of anharmonicity.

Within the harmonic regime, the energy of the CDW amplitude mode can be approximated by that of a harmonic oscillator,

$$E(a) = E_0 + \frac{1}{2} m_{\mathrm{eff}} \omega^2 a^2, \tag{S1}$$

where m_{eff} denotes the effective mass of the phonon mode.

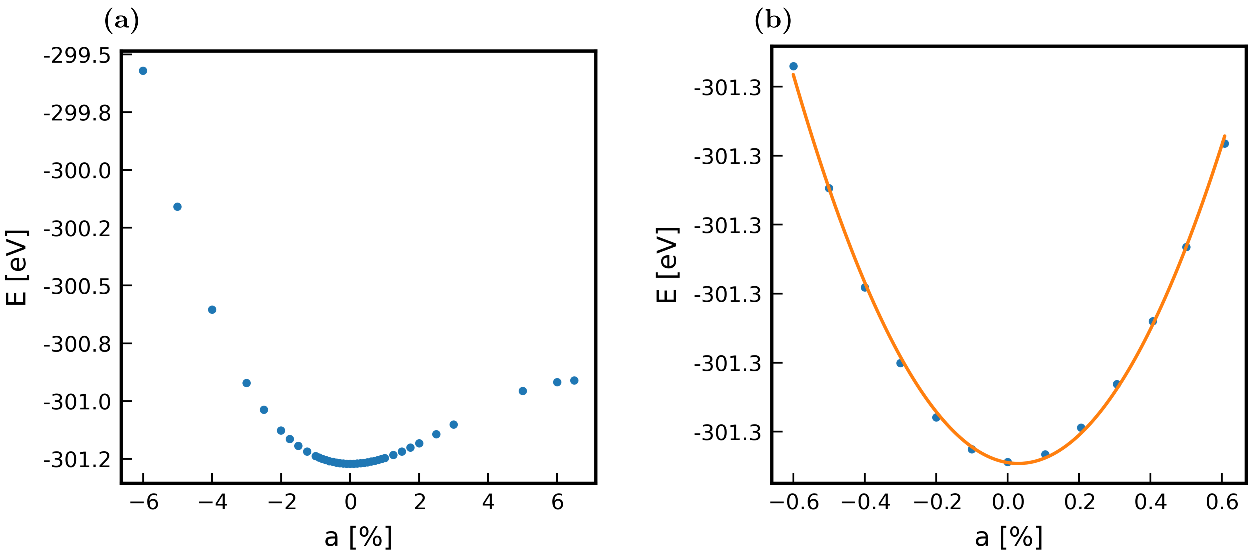

Supplementary Figure S1: (a) DFT total energy as a function of the CDW amplitude a in the range $-6\% \leq a \leq +6\%$. (b) Harmonic (parabolic) fit to the energy curve in the small-amplitude regime.

Taking the second derivative of the total energy with respect to a, $\partial^2 E/\partial a^2$, the phonon frequency can be expressed as

$$\omega = \sqrt{\frac{\partial^2 E/\partial a^2}{m_{\text{eff}}}}. \tag{S2}$$

To extract ω, we perform a parabolic fit to the total energy within the range $|a| < 0.6\%$, as shown in Fig. S1b. This procedure yields $\omega = 2.2$ THz, in reasonably good agreement with the experimental value of 2.4 THz.

III. WANNIERIZATION

Using Wannier90[3,7,8] (version 3.1.0) in library mode interfaced with VASP (version 6.4.3)[3], we constructed a maximally localized Wannier function (MLWF) representing the narrow, half-filled electronic band at the Fermi level. The resulting Wannier orbital exhibits predominantly $d_{3z^2-r^2}$ character. For the Wannier projection, we employed a dense Γ-centered $12 \times 12 \times 12$ k-mesh. Since the correlated band remains fully isolated for all considered CDW amplitudes a, no disentanglement of bands was required.

IV. CRPA CALCULATIONS AND CONVERGENCE TESTS

The cRPA[9–11] calculations were performed in VASP using the following 3 step procedure: (i) a self-consistent DFT ground state calculation, followed by (ii) a one-shot exact-diagonalization step to obtain the unoccupied orbitals and the long-wavelength limit of the polarizability, and (iii) the evaluation of the screened interaction using VASP's internal cRPA implementation. To exclude screening processes within the correlated subspace, we employed the projector method[12].

All three steps of the cRPA procedure in VASP were carried out using a Γ-centered $8 \times 8 \times 1$ k-mesh, a plane-wave energy cutoff of 300 eV, and Gaussian smearing with a width of 0.05 eV. The electronic self-consistency criterion was set to an energy tolerance of 10^{-8} eV. GW-optimized PAW pseudopotentials were employed to include semicore states relevant for screening.

In the exact-diagonalization and cRPA steps, 1024 bands were included to account for a sufficient number of virtual states. The frequency-dependent dielectric matrix was computed (LOPTICS = .TRUE.) to accurately capture the

long-wavelength limit of the polarizability. To improve convergence for the Ta d-states, higher angular components in the charge-density mixing were included up to $l = 4$. The energy cutoff for the Coulomb matrix elements in the cRPA step was set to the converged value of 400 eV.

The screened on-site interaction U and the bare on-site interaction V were systematically converged with respect to the plane-wave cutoff (ENCUT), the number of bands (NBANDS), and the k-mesh. The convergence of U and V with ENCUT is shown in Fig. S3, where 512 bands and a coarse $3 \times 3 \times 1$ k-mesh were used. A final ENCUT value of 300 eV was chosen. Convergence with respect to the k-mesh is presented in Fig. S2, keeping ENCUT = 300 eV and NBANDS = 512. Computational cost reached 44000 core-hours at a $10 \times 10 \times 1$ mesh; therefore, an $8 \times 8 \times 1$ mesh was adopted for the final calculations as a compromise between accuracy and efficiency.

The number of Kohn-Sham orbitals was determined from the convergence of U; V is insensitive to NBANDS, as its value is determined by the bare Coulomb matrix elements. A coarse $3 \times 3 \times 1$ k-grid with ENCUT = 300 eV was used to test NBANDS convergence, as shown in Fig. S4(a). NBANDS = 1024 was chosen for all subsequent calculations. The bare on-site interaction V was further converged with respect to the cutoff for the bare Coulomb matrix elements (VCUTOFF), using an $8 \times 8 \times 1$ k-mesh, ENCUT = 300 eV, and NBANDS = 1024. Convergence is illustrated in Fig. S4(b), and VCUTOFF = 400 eV was adopted as the final value.

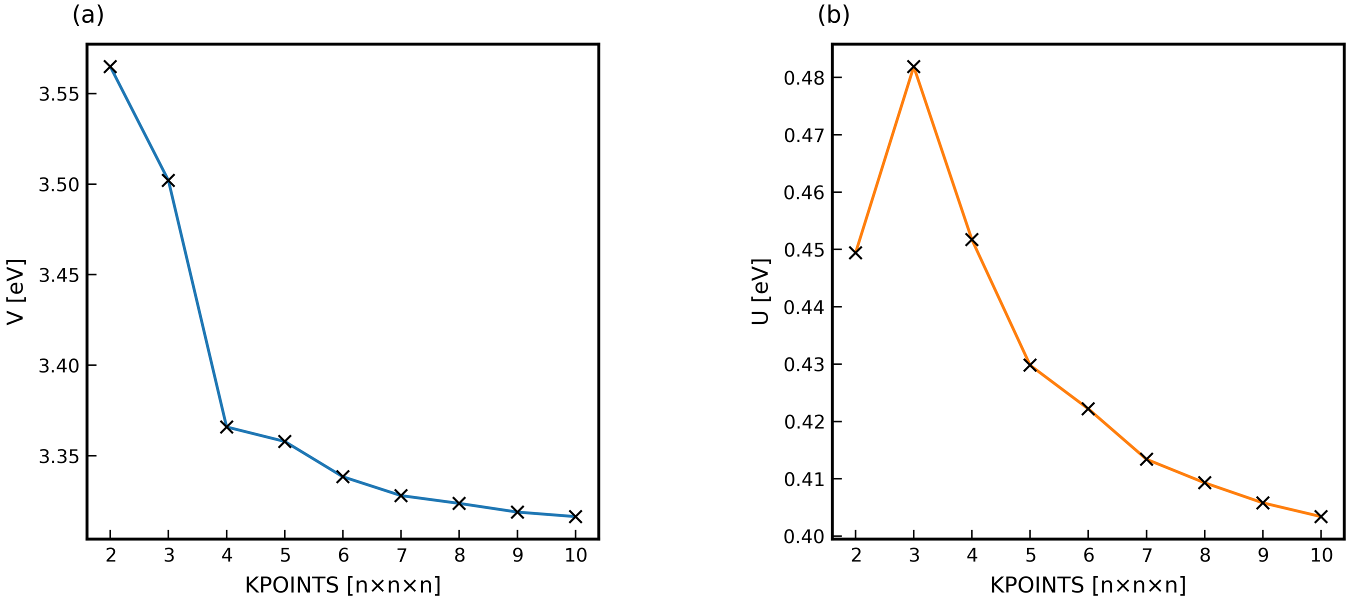

Supplementary Figure S2: Convergence of the (a) bare on-site Coulomb interaction V and (b) screened on-site Coulomb interaction U with respect to the density of the k-mesh (KPOINTS).

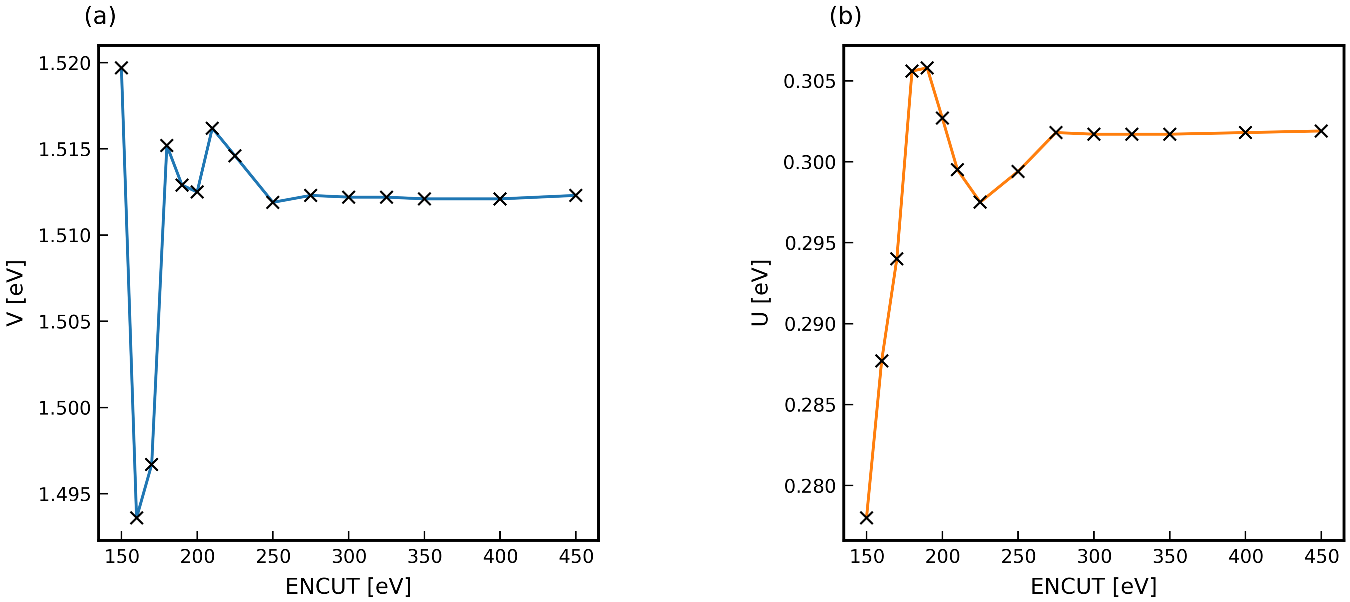

Supplementary Figure S3: Convergence of the (a) bare on-site Coulomb interaction V and (b) screened on-site Coulomb interaction U with respect to the plane-wave energy cutoff (ENCUT).

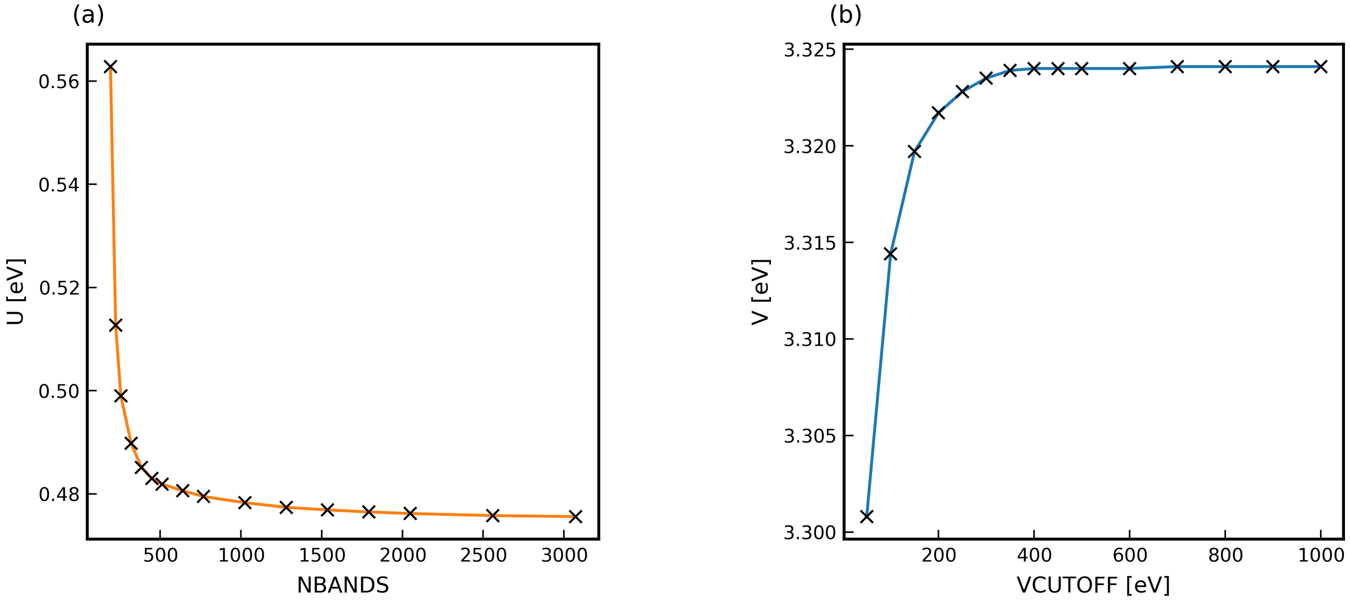

Supplementary Figure S4: Convergence of (a) the screened on-site Coulomb interaction U with respect to the number of Kohn-Sham orbitals (NBANDS) used for screening, and (b) the bare on-site Coulomb interaction V as a function of the energy cutoff for the bare Coulomb matrix elements (VCUTOFF).

V. DMFT CALCULATIONS

The single-band DMFT impurity model was solved using the continuous-time hybridization-expansion quantum Monte Carlo (CT-HYB) impurity solver implemented in w2dynamics.[13] All calculations were performed at an inverse temperature of $\beta = 1200\,\mathrm{eV}^{-1}$. For the CT-HYB solver, we employed 2×10^7 measurement sweeps with 10^7 warmup steps and a decorrelation interval of $NCorr = 50$. Convergence of the DMFT calculations was verified by monitoring the Green's functions and self-energies at each DMFT iteration.

We measured $G(\tau)$ in the Legendre basis to reduce statistical noise and obtain smooth high-frequency behavior.[14] Including more Legendre orders retains more τ-structure and improves the high-frequency tail. But once the noise floor is reached, higher orders mainly introduce statistical noise. Too small orders on the other hand, bias the tail. To determine a suited order for the Legendre polynomials, test runs with different orders were conducted for each CDW amplitude a. The final orders were selected based on visual inspection of the Green's function and self-energy, yielding NLegMax and NLegOrder values between 40 and 70. Stronger CDW distortions (smaller a) typically benefit from higher orders, while weaker distortions (larger a) are adequately captured with 40–50 polynomials. In the case of $a = +2\%$, the Legendre order could be increased beyond 80 without introducing visible noise, consistent with the particularly smooth $G(\tau)$ observed for this CDW amplitude.

VI. ANALYTICAL CONTINUATION

We used the ana_cont package[15] to perform analytical continuations using the maximum entropy method[16,17] with singular value decomposition. The hyperparameter α was determined using the "chi2kink" method[18,19]. Continuations were carried out in two ways: (i) direct analytical continuation of the Green's function $G(i\omega_n)$, and (ii) analytic continuation of the cumulant

$$M(i\omega_n) = \frac{1}{i\omega_n - \mu - \Sigma(i\omega_n)}, \tag{S3}$$

from which the self-energy $\Sigma(\omega)$ on the real-frequency axis is reconstructed as

$$\Sigma(\omega) = \omega + \mu - \frac{1}{M(\omega)}. \tag{S4}$$

The self-energy obtained from the cumulant-based continuation was used to compute the momentum-resolved spectral function $A(k,\omega)$ shown in Fig. 3 of the main text. The momentum-integrated spectral function, $A(\omega) = \sum_{\mathbf{k}} A(\mathbf{k},\omega)$, can then be directly compared to the result obtained from the continuation of $G(i\omega_n)$.

To mitigate unphysical artifacts around the Fermi energy, a slight preblur was applied, with the blur width ranging from 0.0 to 0.0125 depending on the CDW amplitude a. For the computation of the spectral function, a small imaginary $i\eta$ (ranging from 0.008 to 0.012) was added to the denominator of the real-frequency Green's function.

VII. ADDITIONAL RESULTS

In Fig. S5, we show the DFT band structures of (a) the CDW-distorted structure ($a = 0\%$) and (b) the undistorted structure ($a = 6.5\%$). In the absence of the CDW distortion, monolayer 1T-TaS_2 becomes metallic, a phase that is well captured by DFT.

Fig. S6 shows the electronic spectral functions $A(\omega)$ for all considered CDW amplitudes a ranging from $a = -4\%$ to $a = +3\%$.

The total spreads of the maximally localized Wannier functions (MLWFs) for each CDW amplitude a are summarized in Tab. S1. Real-space isosurface plots of the MLWFs were generated and visualized using VESTA[20]. Figure 2 in the main text displays representative isosurfaces for CDW amplitudes between $a = -1\%$ and $a = +2\%$, while Fig. S7 presents the complete set for all considered values of a from $a = -4\%$ to $a = +3\%$. The isosurface levels were adjusted such that each plot corresponds to the same enclosed probability density.

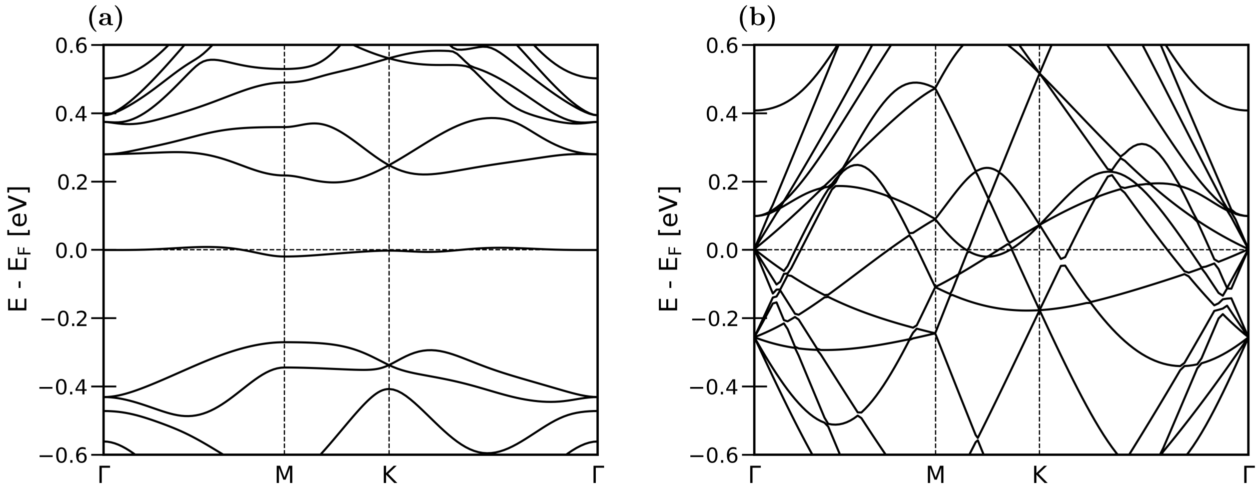

Supplementary Figure S5: DFT band structures of (a) the CDW-distorted structure ($a = 0\%$) and (b) the undistorted structure ($a = 6.5\%$).

Supplementary Table S1: Summary of key quantities across CDW amplitudes a. Ω denotes the Wannier spread, W_b the width of the isolated band, Δ the Mott gap, V the bare on-site Coulomb interaction, U the screened on-site Coulomb interaction, and U/W the ratio between U and W.

a [%]	Ω [Å^2]	W_b [meV]	Δ [eV]	V [eV]	U [eV]	U/W_b
-4.00	13.9	20.6	0.75	4.24	0.79	38.4
-3.00	15.6	21.2	0.69	4.15	0.73	34.2
-2.50	16.9	21.2	—	—	—	—
-2.00	18.7	21.2	0.61	3.99	0.64	30.1
-1.50	21.2	21.0	0.56	3.87	0.59	27.9
-1.00	24.4	20.7	0.50	3.72	0.53	25.6
-0.50	28.9	23.1	0.44	3.54	0.47	20.3
0.00	34.8	27.5	0.38	3.32	0.41	14.8
0.50	42.9	32.9	0.32	3.07	0.35	10.5
1.00	53.9	42.8	0.25	2.79	0.29	6.7
1.50	69.1	56.3	0.17	2.48	0.23	4.1
2.00	95.6	73.1	0.09	2.14	0.18	2.5
2.50	144.5	92.9	—	—	—	—
3.00	207.1	115.4	0.00	1.44	0.11	0.9

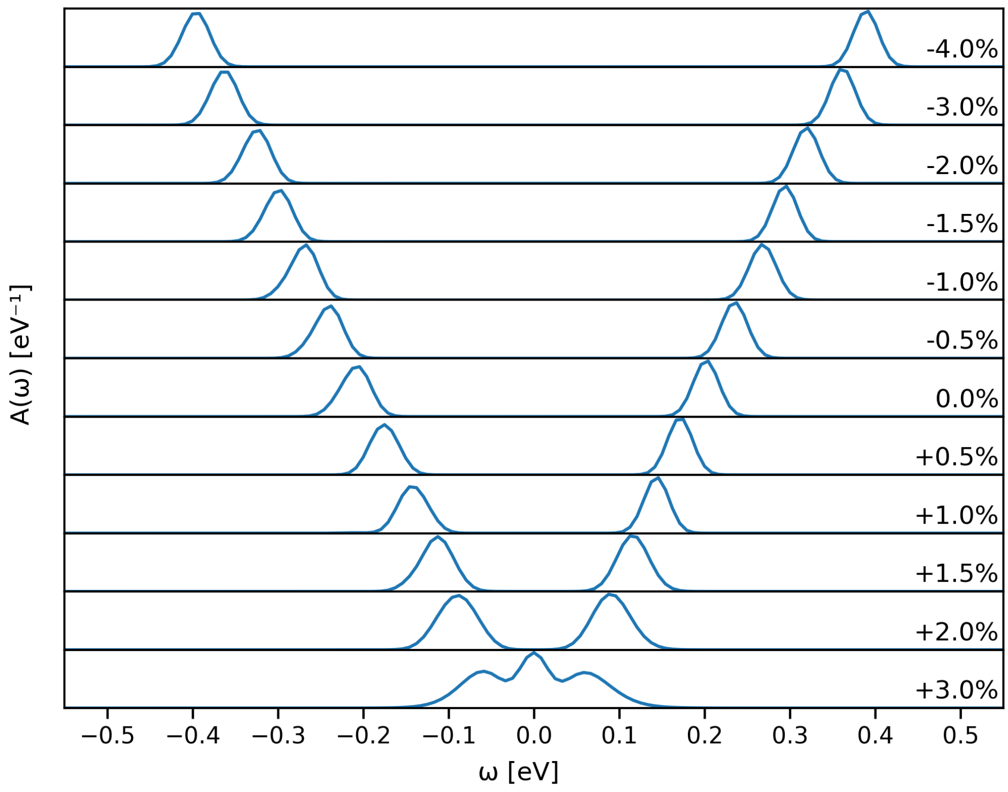

Supplementary Figure S6: Spectral functions $A(\omega)$ for CDW amplitudes a ranging from -4 % to +3 %.

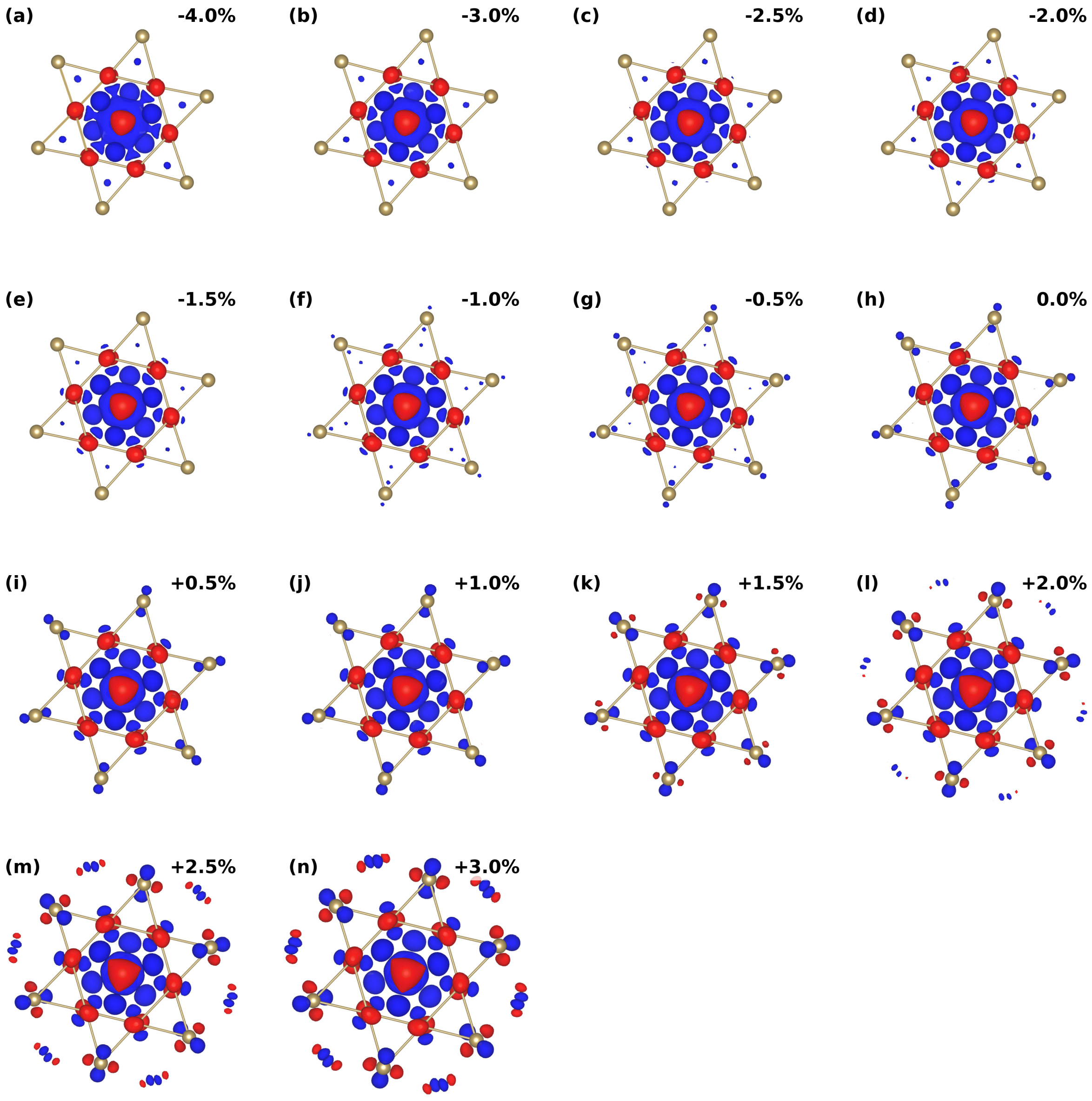

Supplementary Figure S7: Isosurfaces for CDW amplitudes a ranging from (a) -4 % - (n) +3 %.

[1] Kresse, G.; Hafner, J. Ab initio molecular dynamics for liquid metals. *Physical Review B* **1993**, *47*, 558–561.
[2] Kresse, G.; Furthmüller, J. Efficient iterative schemes for ab initio total-energy calculations using a plane-wave basis set. *Physical Review B* **1996**, *54*, 11169–11186.
[3] Kresse, G.; Furthmüller, J. Efficiency of ab-initio total energy calculations for metals and semiconductors using a plane-wave basis set. *Computational Materials Science* **1996**, *6*, 15–50.
[4] Perdew, J. P.; Burke, K.; Ernzerhof, M. Generalized Gradient Approximation Made Simple. *Physical Review Letters* **1996**, *77*, 3865–3868.
[5] Blöchl, P. E. Projector augmented-wave method. *Physical Review B* **1994**, *50*, 17953–17979.
[6] Kresse, G.; Joubert, D. From ultrasoft pseudopotentials to the projector augmented-wave method. *Physical Review B* **1999**, *59*, 1758–1775.
[7] Pizzi, G. et al. Wannier90 as a community code: new features and applications. *Journal of Physics: Condensed Matter* **2020**, *32*, 165902.
[8] Mostofi, A. A.; Yates, J. R.; Lee, Y.-S.; Souza, I.; Vanderbilt, D.; Marzari, N. wannier90: A tool for obtaining maximally-localised Wannier functions. *Computer Physics Communications* **2008**, *178*, 685–699.
[9] Aryasetiawan, F.; Imada, M.; Georges, A.; Kotliar, G.; Biermann, S.; Lichtenstein, A. I. Frequency-dependent local interactions and low-energy effective models from electronic structure calculations. *Phys. Rev. B* **2004**, *70*, 195104.
[10] Kotani, T. Ab initio random-phase-approximation calculation of the frequency-dependent effective interaction between 3d electrons: Ni, Fe, and MnO. *Journal of Physics: Condensed Matter* **2000**, *12*, 2413.
[11] Springer, M.; Aryasetiawan, F. Frequency-dependent screened interaction in Ni within the random-phase approximation. *Phys. Rev. B* **1998**, *57*, 4364–4368.
[12] Kaltak, M. Merging GW with DMFT. *PhD thesis, University of Vienna* **2015**,
[13] Wallerberger, M.; Hausoel, A.; Gunacker, P.; Kowalski, A.; Parragh, N.; Goth, F.; Held, K.; Sangiovanni, G. w2dynamics: Local one- and two-particle quantities from dynamical mean field theory. *Computer Physics Communications* **2019**, *235*, 388–399.
[14] Boehnke, L.; Hafermann, H.; Ferrero, M.; Lechermann, F.; Parcollet, O. Orthogonal polynomial representation of imaginary-time Green's functions. *Physical Review B* **2011**, *84*.
[15] Kaufmann, J.; Held, K. ana_cont: Python package for analytic continuation. *Computer Physics Communications* **2023**, *282*, 108519.
[16] Gubernatis, J. E.; Jarrell, M.; Silver, R. N.; Sivia, D. S. Quantum Monte Carlo simulations and maximum entropy: Dynamics from imaginary-time data. *Physical Review B* **1991**, *44*, 6011–6029.
[17] Sandvik, A. W. Stochastic method for analytic continuation of quantum Monte Carlo data. *Physical Review B* **1998**, *57*, 10287–10290.
[18] Bergeron, D.; Tremblay, A.-M. S. Algorithms for optimized maximum entropy and diagnostic tools for analytic continuation. *Physical Review E* **2016**, *94*.
[19] Kraberger, G. J.; Triebl, R.; Zingl, M.; Aichhorn, M. Maximum entropy formalism for the analytic continuation of matrix-valued Green's functions. *Physical Review B* **2017**, *96*.
[20] Momma, K.; Izumi, F. VESTA 3 for three-dimensional visualization of crystal, volumetric and morphology data. *Journal of Applied Crystallography* **2011**, *44*, 1272–1276.
